# Rovibrational Interaction and Vibrational Constants of the Symmetric Top Molecule ^**14**^NF_**3**_

**DOI:** 10.1155/2013/813249

**Published:** 2013-05-23

**Authors:** Hamid Najib

**Affiliations:** Laboratoire de Physique de la Matière Condensée, Équipe de Spectrométrie Physique, Département de Physique, Université Ibn Tofaïl, Faculté des Sciences, BP 133, 14000 Kénitra, Morocco

## Abstract

Several accurate experimental values of the *α* ^*C*^ and *α* ^*B*^ rotation-vibration interaction parameters and *ω*_*i*_, *x*_*ij*_, and *g*_*ij*_ vibrational constants have been extracted from the most recent high-resolution Fourier transform infrared, millimeter wave, and centimeter wave investigations in the spectra of the oblate symmetric top molecule ^14^NF_3_. The band-centres used are those of the four fundamental, the overtones, the combination, and hot bands identified in the region between 400 cm^−1^ and 2000 cm^−1^. Comparison of our constants with the ones measured previously, by infrared spectroscopy at low resolution, reveals orders of magnitude higher accuracy of the new values. The agreement between our values and those determined by *ab initio* calculations employing the TZ2Pf basis is excellent.

## 1. Introduction

Nitrogen trifluoride NF_3_ is a pyramidal symmetric top molecule belonging to C_3v_ point group. It has four vibrational normal modes:

- two totally symmetric (*A*_1_ type): *ν*_1_ and *ν*_2_;
- two doubly degenerate (*E* type): *ν*_3_ and *ν*_4_.

Today, NF_3_ is employed in the cleaning of the PECVD chambers in the high volume production of liquid crystal displays and silicon-based thin film solar cells. It has been considered as an environmentally preferable substitute for sulphur hexafluoride or perfluorocarbons such as hexafluoroethane [1]. But, recent publications [2–7] reported that NF_3_ is 17,000 times more potent a greenhouse gas than CO_2_ and recommended to include it in the basket of gases controlled by Kyoto protocol and to monitor its environmental levels.

Nitrogen trifluoride is also used in hydrogen fluoride and deuterium fluoride lasers, which are types of chemical lasers.

In spite of these interesting properties and important applications, the spectra of this molecule have not yet been much studied in the past, because of difficulties to obtain high quality spectra and to interpret its perturbed bands. Many of these difficulties have now been overcome through the advent of high-resolution spectrometers and the development of computers.

This is within the framework of this research orientation and the Moroccan/European collaboration that several bands of NF_3_ gas were recently analysed [8–14]. We used independent and complementary methods, Fourier transform infrared (FTIR), millimetre wave (MMW), and centimetre wave (CMW) spectroscopies. All studied IR spectra were recorded at Wuppertal (Germany) with the Bruker 120 HR interferometer, except for the region around 1032 cm^−1^, which was recorded at the University of Giessen (Germany). The rotational spectra were measured in Lille (France) with a computer-controlled MMW spectrometer. The measurements in the CMW range were performed at Kiel (Germany) by means of wave-guide microwave Fourier transform spectroscopy.

The subject of this work is to extract accurate experimental rotation-vibration interaction parameters and anharmonicity constants of the potential function of ^14^NF_3_ from our recent studies by high-resolution spectroscopy and the latest results available in the literature.

## 2. Relationships Linking Vibrational Constants

More recently, we have established the following relationships linking band-centres, harmonic wavenumbers, and anharmonicity constants of a symmetric top molecule [15].

### 2.1. Fundamental Bands *ν*_*i*_ = 1

(1) $$\begin{array}{c} \left(\nu_{i}^{\ell_{i}}\right)=\omega_{i}^{0}+x_{ii}+g_{ii}\ell_{i} \end{array}$$

*ω*_*i*_ is the normal wavenumber of the *i*th mode; *ν*_*i*_ is its vibrational quantum number; *i* refers to a totally symmetric or to a degenerate vibration; *x*_*ii*_ and *g*_*ii*_ are the anharmonicity constants; and *ℓ*_*i*_ is the vibrational angular momentum: |*ℓ*_*i*_ | = *ν*_*i*_, *ν*_*i*_ − 2, …, 1, or 0 only exists for a doubly degenerate vibration.

### 2.2. Harmonic Bands *ν*_*i*_ = *n* (Integer > 1)

(2) $$\begin{array}{c} \left(n\nu_{i}^{\ell_{i}}\right)=n{\left(\nu_{i}^{\pm 1}\right)}^{0}+\left(n^{2}-n\right)x_{ii}+\left(\ell_{i}^{2}-n\right)g_{ii}. \end{array}$$

### 2.3. Combination Bands *ν*_*i*_ = *n* and *ν*_*i*′_ = *m* (Integer), *i* ≠ *i*′

(3) (nνiℓi+mνi′ℓi′)0=(nνiℓi)0+(mνi′ℓi′)0+nmxii′+gii′ℓiℓi′.

For the oblate symmetric top NF_3_, we count 4*ω*_*i*_^0^, 10*x*_*ij*_, and 3*g*_*ij*_; *i* ≤ *j*.

## 3. The Ground State Constants of ^14^NF_3_

The experimental ground state (GS) axial rotational parameters for the symmetric top molecule ^14^NF_3_ employed in the present study and reported in Table 1 come from different sources. 

For the *C*_0_ *K*-dependent constant, we used in our high-resolution studies the following values:

- *C*_0_ = 0.1949980 (10): a preliminary value obtained in the course of our work on the 2*ν*_4_ overtone band of ^14^NF_3_,
- *C*_0_ = 0.19499250 (44): the definitive value adopted in the study of the *ν*_4_ = 2 state of ^14^NF_3_. In the two cases, we used the “*loop method*” as described in [16].

For the *B*_0_ *J*-dependent constant, the values adopted were determined by Höhe et al. [17], Breidung et al. [18], and Cazzoli and Puzzarini [19].

## 4. The Rovibrational Bands of ^14^NF_3_ Observed below 2000 cm^−1^

### 4.1. The *ν*_1_ Fundamental Band [17]

The *ν*_1_ = 1 excited state of ^14^NF_3_ was investigated by FTIR spectroscopy and with saturation technique using CO_2_ side bands as a tunable infrared source. This level was considered unperturbed, and the fit gave the band-centre: (*ν*_1_)^0^ = 1032.00123750 (47) cm^−1^. For the excited rotational constants, the values were determined relative to the GS.

### 4.2. The *ν*_4_ Fundamental Band [14]

The lowest fundamental band *ν*_4_ of the nitrogen trifluoride ^14^NF_3_ was studied by high-resolution FTIR, MMW, and CMW spectroscopies near 493 cm^−1^. The analysis of the *ν*_4_ = 1 state included *ℓ*(2, 2), *ℓ*(2, − 1), *ℓ*(2, − 4), and *k*(0, 6) intravibrational interactions and gave the band-centre: (*ν*_4_)^0^ = 493.4227759 (89) cm^−1^.

### 4.3. The *ν*_2_ Fundamental, 2*ν*_2_ − *ν*_2_ Hot, 2*ν*_2_ Overtone, and *ν*_2_ + *ν*_4_ Combination Bands [13]

They were analysed by FTIR and MMW spectroscopies. The corresponding excited states were treated as isolated levels, and the following band-centres were determined:

(4) $$\begin{array}{c} {\left(\nu_{2}\right)}^{0}=647.1340617\;\left(73\right)\;{\text{cm}}^{-1},\\ {\left(2\nu_{2}-\nu_{2}\right)}^{0}=645.121943\;\left(14\right)\;{\text{cm}}^{-1},\\ {\left(\nu_{2}+\nu_{4}\right)}^{0}=1138.276629\;\left(10\right)\;{\text{cm}}^{-1}. \end{array}$$

Because of the low intensity of the 2*ν*_2_ overtone, the *ν*_2_ = 2 state constants were determined from the 2*ν*_2_ − *ν*_2_ hot and *ν*_2_ fundamental bands.

We point out that any explanation was offered for a severe intensity perturbation observed in the spectra of *ν*_2_ and 2*ν*_2_ bands (see [13]).

### 4.4. The *ν*_3_ Fundamental Band [12]

The perpendicular band *ν*_3_ of the symmetric top ^14^NF_3_ was analysed by high-resolution FTIR and MMW spectroscopies. The *ν*_3_ = 1 excited state was considered vibrationally isolated, but several intravibrational interactions were included in the final fit. The band-centre obtained is (*ν*_3_)^0^ = 907.5413300 (72) cm^−1^.

### 4.5. The 2*ν*_4_ Overtone Band [11]

The *ν*_4_ = 2 excited state of the oblate molecule ^14^NF_3_, lying near 985 cm^−1^, was successfully studied by high-resolution FTIR and MMW spectroscopies. Assumed to be isolated, the treatment of this level gave the following band-centres for the parallel and perpendicular components:

(5) $$\begin{array}{c} {\left(2\nu_{4}^{0}\right)}^{0}=983.701767\;\left(34\right)\;{\text{cm}}^{-1},\\ {\left(2\nu_{4}^{\pm 2}\right)}^{0}=986.622364\;\left(18\right)\;{\text{cm}}^{-1}. \end{array}$$

### 4.6. The 2*ν*_3_ Overtone and *ν*_1_ + *ν*_3_ Combination Bands [10]

In the analysis of these bands, we used spectra recorded by Fourier transform spectrometer. The same models applied to the *ν*_4_ = 2 and *ν*_3_ = 1 excited states were applied to treat the 2*ν*_3_ and *ν*_1_ + *ν*_3_ bands. We obtained the following results:

(6) $$\begin{array}{c} {\left(2\nu_{3}^{0}\right)}^{0}=1803.130213\;\left(57\right)\;{\text{cm}}^{-1},\\ {\left(2\nu_{3}^{\pm 2}\right)}^{0}=1810.423993\;\left(21\right)\;{\text{cm}}^{-1},\\ {\left(\nu_{1}+\nu_{3}\right)}^{0}=1931.577516\;\left(19\right)\;{\text{cm}}^{-1}. \end{array}$$

### 4.7. The *ν*_1_ + *ν*_4_ Combination Band [8, 9]

The *ν*_1_ + *ν*_4_ perpendicular band of ^14^NF_3_ was studied by high-resolution infrared spectroscopy. It was treated by a model taking into account *ℓ*- and *k*-type intervibrational interactions. The band-centre obtained in the *D*-reduction of the rovibrational Hamiltonian is (*ν*_1_+*ν*_4_)^0^ = 1523.040783 (34) cm^−1^.

## 5. Results and Discussion

In Table 2, we gather the recent measurements, at high-resolution, of the centres of the all studied bands between 400 cm^−1^ and 2000 cm^−1^ and the corresponding experimental rotation-vibration interaction constants: *α* ^*C*^ = *C*_0_ − *C*′ and *α* ^*B*^ = *B*_0_ − *B*′ and *C*′ and *B*′ being the constants of the upper level.

The values of the rovibrational interaction constants were determined by *ab initio* calculations (Table 2) for only the fundamental bands. The agreement with our values is excellent. 

Equation (2) leads to *x*_24_ = (*ν*_2_+*ν*_4_)^0^ − (*ν*_2_)^0^ − (*ν*_4_)^0^ = −2.280208 (11) cm^−1^.

For the 2*ν*_4_ harmonic band, we obtain the system of equations:

(7) $$\begin{array}{c} {\left(2\nu_{4}^{0}\right)}^{0}=2{\left(\nu_{4}^{\pm 1}\right)}^{0}+2x_{44}-2g_{44},\\ {\left(2\nu_{4}^{\pm 2}\right)}^{0}=2{\left(\nu_{4}^{\pm 1}\right)}^{0}+2x_{44}+2g_{44}. \end{array}$$

We derive *x*_44_ = −0.841743 (22) cm^−1^ and *g*_44_ = 0.730149 (21) cm^−1^.

As for 2*ν*_4_, we deduce *x*_33_ = −4.15278 (18) cm^−1^ and *g*_33_ = 1.82344 (18) cm^−1^.

Using the band-centres of *ν*_1_ and *ν*_4_, we obtain *x*_14_ = −2.383248 (49) cm^−1^. We can also derive, from the band-centres of *ν*_1_ and *ν*_3_, the anharmonic constant: *x*_13_ = −7.965051 (26) cm^−1^.

All experimental anharmonicity constants determined in this work for the nitrogen trifluoride ^14^NF_3_ are given in Table 3.

The experimental centre of the 2*ν*_2_ band was estimated from the ^*Q*^ *Q* branches edge at 1292.26 cm^−1^. But a precise value can be obtained combining the values of the experimental centres of *ν*_2_ and 2*ν*_2_ − *ν*_2_ (Table 2). From the relationships:

(8) $$\begin{array}{c} {\left(2\nu_{2}\right)}^{0}=2{\left(\nu_{2}\right)}^{0}+2x_{22},\\ {\left(2\nu_{2}-\nu_{2}\right)}^{0}={\left(\nu_{2}\right)}^{0}+2x_{22}, \end{array}$$

we can indeed deduce that (2*ν*_2_)^0^ = 1292.25004 (21) cm^−1^ and *x*_22_ = −1.006059 (11).

The values obtained for *x*_22_, *x*_44_, and *g*_44_ are small suggesting that the corresponding levels *ν*_2_ = 2 and *ν*_4_ = 2 are not significantly affected by the anharmonic perturbations, whereas the vibrational dependence is extremely important for the *ν*_1_ = *ν*_3_ = 1 and *ν*_2_ = *ν*_3_ = 1 levels.

It is worth noting that our values of the anharmonicity constants of ^14^NF_3_ are in good agreement with previous medium experimental studies (column 4 of Table 3), but significantly more accurate by at least three orders of magnitude.

One can notice the fair agreement between our values and those obtained by *ab initio* methods [18] employing the TZ2Pf basis.

We can also extract accurate values of the harmonic wavenumbers of the oblate symmetric top molecule ^14^NF_3_. The results are gathered in Table 4.

The agreement between our values and those determined by the theoretical *ab initio* calculations is significantly worse. We think that it is necessary to give much more credibility to our *ω*_*i*_^0^ values which were deduced from experimental constants.

## 6. Conclusion

Using the recent accurate results obtained for the bands below 2000 cm^−1^, of the symmetric top molecule ^14^NF_3_, several rovibrational interaction and anharmonicity constants have been deduced.

Our results contribute incontestably to the experimental knowledge of the molecular potential of this molecule which helps to test and improve theoretical models.

## Figures and Tables

**Table 1:** Ground state constants (cm^−1^) of  ^14^NF_3_.

Parameter	Value [12, 17]	Value [11, 18]	Value [11, 19]
*C*_0_	0.1949980 (10)	0.19499250 (44)	0.19499250 (44)
*B*_0_	0.3562828965 (40)	0.3562827950 (15)	0.3562828891 (21)

Numbers in parentheses represent one standard deviation in units of the last digit quoted.

**Table 2:** Band-centres and rovibrational interaction constants of  ^14^NF_3_ molecule (in cm^−1^).

Band or component	Experimental band-centre, this work	Experimental *α* ^*C*^ × 10^3^, this work	*Ab initio α* ^*B*^ × 10^3^, [18]	Experimental *α* ^*B*^ × 10^3^, this work	*Ab initio α* ^*B*^ × 10^3^, [18]
*ν*_1_	1032.00123750 (47)	0.593526 (40)	0.643	−1.4496825 (43)	−1.227
*ν*_2_	647.1340617 (73)	0.4346 (10)	0.404	1.289844 (18)	1.300
*ν*_3_	907.5413300 (72)	0.7374 (11)	0.857	2.628451 (22)	2.758
*ν*_4_	493.4227759 (89)	0.5566 (10)	0.544	0.150021 (23)	0.164
2*ν*_2_ − *ν*_2_	645.121943 (14)	0.8777 (10)	*— *	2.592650 (37)	*— *
2*ν*_2_	1292.25004 (21)^a^	0.8777 (10)	*— *	2.592650 (37)	*— *
*ν*_2_ + *ν*_4_	1138.276629 (10)	0.9922 (10)	*— *	1.447060 (43)	*— *
2*ν*_4_^0^	983.701767 (34)	1.10135 (63)	*— *	0.192090 (23)	*— *
2*ν*_4_^±2^	986.622364 (18)	1.10101 (56)	*— *	0.302179 (50)	*— *
2*ν*_3_^0^	1803.130213 (57)	1.47497 (80)	*— *	4.84308 (22)	*— *
2*ν*_3_^±2^	1810.423993 (21)	1.49747 (50)	*— *	5.24715 (12)	*— *
*ν*_1_ + *ν*_3_	1931.577516 (19)	1.30958 (56)	*— *	1.460127 (78)	*— *
*ν*_1_ + *ν*_4_	1523.040783 (34)	1.37339 (84)	*— *	−1.13109 (44)	*— *
*ν*_2_ + *ν*_3_	1546.30^b^	—	—	—	*— *

Numbers in parentheses represent one standard deviation in units of the last digit quoted.

^a^Value extracted from the values of (*ν*_2_)^0^ and *x*_22_ (Tables 2 and 3). ^b^Value estimated from our FTIR spectrum which is in course of study.

**Table 3:** Anharmonicity constants of  ^14^NF_3_ (in cm^−1^).

Anharmonicity constant	Experimental value, this work	*Ab initio* TZ2Pf [18]	Experimental value [20, 21]
*x*_11_	—	−3.15	−2.9
*x*_12_	—	−3.56	−4
*x*_13_	−7.965051 (26)	−8.14	−9.9
*x*_14_	−2.383248 (49)	−2.08	−1.5
*x*_22_	−1.006059 (11)	−0.88	—
*x*_23_	−8.53^a^	−6.46	−7.6
*x*_24_	−2.280208 (11)	−1.98	−2.5
*x*_33_	−4.15278 (18)	−4.19	—
*x*_34_	—	−5.12	—
*x*_44_	−0.841743 (22)	−0.79	−0.820
*g*_33_	1.82344 (18)	1.96	—
*g*_34_	—	−0.10	—
*g*_44_	0.730149 (22)	0.72	0.729
*x*_34_ + *g*_34_	—	—	−1

Numbers in parentheses are one standard deviation in units of the last digit quoted.

^a^Value deduced from the (*ν*_2_ + *ν*_3_)^0^ given in Table 2.

**Table 4:** Harmonic wavenumber constants of  ^14^NF_3_ (in cm^−1^).

Harmonic wavenumber	Experimental value, this work	*Ab initio* unscaled value, [22]	*Ab initio *value TZ2Pf, [18]
*ω*_1_^0^	—	1042.8	1053.8
*ω*_2_^0^	648.140120 (18)	659.3	660.7
*ω*_3_^0^	909.84264 (43)	905.0	935.7
*ω*_4_^0^	493.534359 (52)	500.6	502.8

Numbers in parentheses represent one standard deviation in units of the last digit quoted.
